# La grossesse en hémodialyse chronique: à propos de 25 cas survenus dans le Sud Tunisien

**DOI:** 10.11604/pamj.2020.36.195.20514

**Published:** 2020-07-20

**Authors:** Hanen Chaker, Slim Masmoudi, Salma Toumi, Najla Dammak, Jamil Hachicha, Khawla Kammoun, Soumaya Yaich, Mohamed Ben Hmida

**Affiliations:** 1 Unité de Recherche de Pathologie Rénale, Service de Néphrologie, Faculté de Médecine, CHU Hedi Chaker, Sfax, Tunisie

**Keywords:** Grossesse, hémodialyse, insuffisance rénale chronique terminale, complications materno-fœtales, Pregnancy, hemodialysis, terminal chronic renal failure, materno-fetal complications

## Abstract

**Introduction:**

la survenue de grossesse au cours de l’hémodialyse chronique est rare. Néanmoins, avec l’évolution des techniques de dialyse, une amélioration de la fertilité est possible. Le but de notre travail est de rapporter notre expérience concernant la survenue d'une grossesse chez les patientes dialysées et de dégager les facteurs impliqués dans sa réussite.

**Méthodes:**

notre étude rétrospective a porté sur 25 grossesses spontanées survenues chez 19 patientes traitées par hémodialyse périodique dans différents centres d’hémodialyse du sud tunisien sur une période de 34 ans.

**Résultats:**

l’âge maternel de survenue de grossesse était en moyenne égal à 35,6 ans [23-44ans] avec une ancienneté moyenne en hémodialyse de 4,22 ans [1-17 ans]. Sept patientes (37%) avaient une diurèse résiduelle (>500 ml/24h). Le volume horaire hebdomadaire de dialyse était ≥16 heures par semaine dans 7 cas et ≥20 heures dans 4 cas. Le succès de la grossesse (nouveau-né survivant au moins 28 jours) était estimé à 56%. L’âge gestationnel médian était de 34 semaines d’aménorrhée [28-38 SA]. Le poids néonatal moyen est égal à 1970g [1500g-2300g]. L’étude analytique a montré une corrélation significative entre l’augmentation des heures de dialyse par semaine et la réussite de la grossesse (R= 0,59; p= 0,002).

**Conclusion:**

on souligne qu’avec une prise en charge adéquate et notamment la majoration du nombre de séances de dialyse, les complications materno-fœtales peuvent être minimisées et la balance risque-bénéfice vire vers donner la chance à une femme dialysée de devenir maman.

## Abstract (en)

**Introduction:**

the occurrence of pregnancy in patients on chronic haemodialysis is rare. However, given the evolution in dialysis technique, improvement in fertility is possible. The purpose of our study was to report our experience concerning the occurrence of pregnancy in patients on dialysis and to identify factors involved in its success.

**Methods:**

we conducted a retrospective study on 25 spontaneous pregnancies occurred in 19 patients treated with periodical hemodialysis in different hemodialysis centers in the south of Tunisia over a period of 34 years.

**Results:**

maternal age at the onset of pregnancy was, on average, 35.6 years [23-44 years] with an average seniority in hemodialysis of 4.22 years [1-17 years]. Seven patients (37%) had residual diuresis (>500 ml/24h). The prescribed weekly number of hours of dialysis was ≥16 hours per week in 7 cases and ≥20 hours in 4 cases. Success of pregnancy (new-born surviving at least 28 days) was estimated at 56%. The median gestational age was 34 weeks of amenorrhea [28-38 WA]. The average neonatal weight was equal to 1970g [1500g-2300g]. Analytical study showed a significant correlation between the increase in the hours of dialysis per week and the success of pregnancy (R=0.59; p=0.002).

**Conclusion:**

it was noted that with adequate support and in particular, increasing the number of sessions of dialysis, materno-fetal complications can be minimized and the balance risk-benefit can turn the chance for a woman on dyalisis to become pregnant.

## Introduction

La conception en hémodialyse (HD) est un évènement rare et à haut risque materno-fœtal. En effet, les perturbations du métabolisme et de la volémie interfèrent avec le fonctionnement normal de l’axe hypothalamo-hypophysaire entrainant des cycles anovulatoires, une aménorrhée et une diminution de la libido. Vu la rareté de cet évènement et le risque de complications maternelles et fœtales, la grossesse en insuffisance rénale chronique (IRC) au stade d’HD était longtemps considérée comme une situation stressante aussi bien pour les néphrologues que pour les obstétriciens. Généralement, l’espoir de conception pour les femmes en âge de procréation est lié à une éventuelle greffe rénale. Cette perception pessimiste est le résultat du manque d’études sur les grossesses en HD. L’Association Européenne de Dialyse et Transplantation a déclaré que seulement 23% de grossesses ont abouti durant la période allant de 1967 à 1978 [1]. La description de la première grossesse suivie d’un accouchement à terme d’un enfant vivant chez une patiente en HD publiée par Confortini en 1971 [2]. L’évolution des techniques d’hémodialyse et la prise en charge précoce des complications ont permis d’améliorer nettement le pronostic; outre la collaboration étroite entre néphrologue et gynécologue. Nous rapportons dans ce travail 25 grossesses spontanées survenues chez 19 patientes traitées par hémodialyse périodique dans les centres de dialyse du sud Tunisien sur une période de 34 ans.

## Méthodes

Les données étaient colligées des différents centres après contact téléphonique. Une fiche a été envoyée par courrier électronique aux différents médecins responsables des centres de dialyse. Les paramètres à relever étaient l’âge, les antécédents gynécologiques, (avortement, de gestation et de parité), la néphropathie initiale, la fréquence et la durée des séances d’hémodialyse par semaine; l’examen clinique incluant la mesure de la pression artérielle (PA) et la diurèse résiduelle, la biologie incluant le taux d’urée et le taux d’hémoglobine; le délai de survenue de la grossesse par rapport au début du traitement par hémodialyse périodique, la circonstance de découverte de la grossesse, l’âge de la grossesse au moment de sa découverte. Outre ces paramètres, le suivi de la patiente a été précisé; basé sur la mesure de la PA, le dosage du taux d’hémoglobine, le dosage du taux d’urée, la recherche des complications maternelles liées à la grossesse (pré-éclampsie, éclampsie, HELLP syndrome: hemolysis, elevated liver enzymes, low platelet count). La surveillance du fœtus a été précisée (perception des mouvements actifs, échographie obstétricale) à la recherche de complications fœtales à type de retard de croissance intra utérin (RCIU), ou hydramnios; ainsi que les circonstances de l’accouchement (le terme de l’accouchement, accouchement par voie basse ou césarienne; l’examen du nouveau-né (score d’Apgar à la naissance, poids de naissance, hospitalisation au service de néonatologie) ont été aussi notés. L’analyse statistique a été réalisée avec le logiciel: Statistical Package for Social Science (SPSS) de Windows (version 20.0). Les variables qualitatives sont représentées en effectifs et en pourcentages. Les variables quantitatives sont représentées en moyennes ± écarts types pour les variables à distribution gaussienne et en médiane (minimum, maximum) pour les variables à distribution non gaussienne. Vu la faible taille de l’échantillon, nous avons utilisé les tests non paramétriques (coefficients de corrélation de Spearman et le test de Mann-Whitney). Le seuil de 0,05 a été utilisé pour juger la significativité du test.

## Résultats

L’âge maternel de survenue de grossesse était en moyenne égal à 35,6 ans [23 - 44ans] avec une ancienneté moyenne en hémodialyse de 4,22 ans [1 - 17ans]. La néphropathie initiale était indéterminée dans 14 cas (soit 74% des cas), une néphropathie interstitielle chronique (NIC) dans 3 cas (soit 16% des cas), une néphropathie lupique dans un cas (soit 5% des cas) et une hyalinose segmentaire focale (HSF) dans un cas (soit 5% des cas). L’hypertension artérielle était présente chez 7 patientes (37%). Aucune patiente n’était diabétique au moment de la conception. Sept patientes (37%) avaient une diurèse résiduelle (> 500ml/24h) (Tableau 1). Le taux moyen d’hémoglobine était à 8 ± 1,54g/dl [5,1 - 10,6g/dl] avec recours à l’érythropoïétine dans 12 cas (48%), aux folates dans 11 cas (44%), au fer en intraveineux dans 11 cas (44%) et à la transfusion dans 6 cas (24%). Le taux moyen d’urée est égal à 22,19 ± 4,29mmol/L [14,66 - 28mmol/L]. Chez toutes nos patientes la grossesse était inattendue, et aucune d’entre elles n’utilisait un moyen de contraception. L’âge moyen de la grossesse au moment de son diagnostic était de 14 semaines d’aménorrhée (SA) [3 - 30 SA] (Tableau 2). La découverte de la grossesse a été faite avant 12 SA dans 14 grossesses (56%), après 12 SA dans 8 grossesses (32%) (tardive dans 3 cas à 25, 29 et 30 SA), le terme était inconnu dans 3 cas (12%). Le diagnostic de la grossesse a été suspecté devant un retard des menstruations dans 7 cas (28%), des signes sympathiques de la grossesse dans 6 cas (24%) et une augmentation du volume abdominal dans 2 cas (8%). Le diagnostic a été fait dans 9 cas (36%) par dosage des β-hCG, dans 9 cas par échographie seule (36%), par les deux dans 1 cas (4%) et non précisé dans 6 cas (24%).

**Tableau 1:** données épidémiologiques

Variables	Patientes
Nombre de patientes	19
Nombre de grossesses	25
Age médian à la conception, années	35,63 ± 5,62
Patientes âgées de 20 à 25 ans à la conception, n (%)	1(4%)
Patientes âgées de 26 à 30 ans à la conception, n (%)	2(8%)
Patientes âgées de 31 à 35 ans à la conception, n (%)	7(28%)
Patientes âgées de 36 à 40 ans à la conception, n (%)	10(40%)
Patientes âgées de 41 à 45 ans à la conception, n (%)	5(20%)
Temps médian du début de la dialyse à la conception, années	4,56 ans ± 3,55
Diurèse conservée, n (%)	7(37%)
**Néphropathie initiale, n (%)**	
Indéterminée	14(74%)
Néphropathie interstitielle chronique (NIC)	3(16%)
Néphropathie lupique	1(5%)
Une hyalinose segmentaire focale (HSF)	1(5%)
HTA préexistante, n (%)	7(37%)
Diabète, n (%)	0

**Tableau 2:** données maternelles

Nom	Gestité	Parité	Age de survenue de la grossesse	Néphropathie initiale	Diagnostic de grossesse (SA)	Diurèse (ml)	Hb (g/dl)
NS	2	2	26	indéterminée	13	500	6
CA	3	3	35	indéterminée	11	0	8
BD	6	5	39	NIC	10	0	8,2
KD	2	1	28	indéterminée	7	0	8
AH	2	2	23	indéterminée	6	0	9
AM 1	2	2	36	indéterminée	-	-	-
AM 2	3	3	43	indéterminée	12	-	-
JH	2	3	31	lupique	5	0	9
AA 1	9	6	42	indéterminée	3	0	8
AA 2	10	7	44	indéterminée	9	0	10,6
KS	1	1	33	indéterminée	16	0	9
NK 1	4	4	37	indéterminée	20	0	5,1
NK 2	5	5	39	indéterminée	10	1000	7,6
WB	7	5	36	indéterminée	20	0	9,4
MW	6	4	26	indéterminée	20	0	9
RA	7	5	33	indéterminée	29	500	5,8
LD	4	2	35	NIC	6	500	9,1
NM	4	3	36	HSF	25	500	8,5
Aam	-	-	32	indéterminée	12	500	5,2
XY	4	3	43	indéterminée	-	-	-
FS	7	2	40	indéterminée	-	-	-
SG	5	5	41	NIC	30	1000	9

Le volume horaire hebdomadaire de dialyse était ≥16 heures par semaine dans 7 cas et ≥20 heures dans 4 cas. Le succès de la grossesse (nouveau-né survivant au moins 28 jours) était estimé à 56%. L’âge gestationnel médian était de 34 semaines d’aménorrhée [28 - 38 SA]. Le poids néonatal moyen est égal 1970g [1500g - 2300g]. L’étude analytique a montré une corrélation significative entre l’augmentation des heures de dialyse par semaine et la réussite de la grossesse (R=0,59; p=0,002). Parmi les complications maternelles, l’hypertension artérielle était la complication la plus fréquemment retrouvée (7 cas sur 20 si on élimine les 4 avortements précoces et l’interruption volontaire de grossesse (IVG), soit 35% des cas). A noter que 43% des patientes avaient une HTA préexistante à la grossesse. Les chiffres tensionnels étaient bien contrôlés sous traitement antihypertenseur. Par ailleurs, aucun cas de pré-éclampsie, de diabète gestationnel ni de HELLP syndrome n’a été noté. Parmi les complications obstétricales, on a recensé 2 cas d’hémorragie de la délivrance. On n’a pas noté de complications de l’abord vasculaire à type de sténose ou d’infection au niveau de la fistule artério-veineuse. Parmi les complications fœtales, la prématurité était fréquente, présente dans 15 cas (60%): 10 cas (40%) de prématurité moyenne, 4 cas (16%) de grande prématurité et un cas (4%) de prématurité extrême (Tableau 3). Parmi les autres complications, l’hydramnios a été retrouvé dans 3 cas (16%). Le retard de croissance intra-utérin a été retrouvé dans 13 cas (52%), si on considère la définition de l’OMS (poids < 2500g quel que soit le terme). Six cas de décès (24%) ont été décrits, dont 4 morts fœtales in utero (MFIU) et 2 décès néonataux survenus dans les premiers jours après la naissance (à H12 et H24).

**Tableau 3:** données obstétricales

Variables	Résultats
Naissances vivantes, n(%)	16(64%)
Grossesse à terme, n(%)	2(8%)
**Prématurité, n(%)**	
Total	15(60%)
Moyenne	10(40%)
Grande prématurité	4(16%)
Extrême	1(4%)
RCIU, n (%)	13(52%)
MFIU, n (%)	4(16%)
Mort néonatale (< 28j de vie), n (%)	2(8%)
Hydramnios, n (%)	3(12%)
**Poids de naissance, n (%)**	
> ou =2500 g	0
1500 < PDN < 2500 g	11(44%)
< ou = 1500	2(8%)
Données manquantes	12(48%)

## Discussion

Dans notre étude, l’âge maternel de survenue de grossesse était en moyenne égal à 35,63 ans ± 5,62 (entre 23 et 44 ans). Il est supérieur à celui de la littérature qui est de 33 ans avec des extrêmes allant de 18 ans à 43 ans [3]. Les patientes en âge présumé de procréation (âge inférieur à 50 ans) représentent une proportion non négligeable de l’ensemble des hémodialysées à prendre en considération et à surveiller. Le néphrologue doit garder à l’esprit l’éventualité d’une grossesse chez ces patientes pour la guetter à temps et prendre les mesures nécessaires pour optimiser le déroulement de cet évènement à la fois rare et attendu avec impatience chez ces femmes. En effet, l’existence d’une maladie rénale chronique peut être associée à une augmentation de la morbi-mortalité fœtale et maternelle lors d’une grossesse et dans le post-partum. Idéalement, ces risques devraient être évalués et discutés avant la planification d’une grossesse chez des patientes connues pour des néphropathies chroniques [4] surtout qu’il existe un risque accru de précipiter la néphropathie vers le stade terminal. La question se pose autrement en cas d’insuffisance rénale en hémodialyse chronique puisque l’insuffisance rénale est déjà terminale; les enjeux sont essentiellement liés aux possibles complications maternelles et/ou fœtales due à l’urémie. Quel que soit la néphropathie l’attitude du néphrologue sera identique entre autre une optimisation de la dialyse. Néanmoins une attention particulière sera apportée en cas de diabète vu les possibles complications conséquentes [5].

En présence de gros reins polykystiques, les lombalgies seraient plus fréquentes. En cas de maladie lupique, la grossesse est connue comme un facteur déclenchant d’une poussée de la maladie bien que généralement la maladie lupique est éteinte en dialyse. Dans notre série, on n’a pas noté une aggravation ou une poussée de la maladie lupique. Concernant la durée moyenne d’hémodialyse avant la conception, elle était de 4,22 ans (1-17ans) alors que dans la littérature elle est de 5,5 ans (0-18ans) dans la méta analyse de Piccoli publiée en 2016 [6]. La diurèse résiduelle était présente chez sept patientes (37%): un litre par 24h dans 2 cas et de 500ml par 24h dans les autres cas. Elle n’était pas associée dans notre série à la réussite de la grossesse. L’anémie était présente chez toutes les patientes avant la grossesse avec un taux moyen d’hémoglobine à 8 ± 1,54g/dl (5,1 - 10,6g/dl) avec recours à l’érythropoïétine dans 12 cas et au fer en intraveineux dans 11 cas; ce qui a limité le recours à la transfusion (6 cas). L’objectif d’hémoglobine chez la femme enceinte dialysée est de 10-11g/dl [7]. La transfusion doit être évitée car source d’allo-immunisation en particulier chez la femme enceinte [8]. Concernant l’utilisation d’EPO pendant la grossesse, il n’est pas retrouvé de récepteur à l’EPO dans le placenta humain ni de complications secondaires à son utilisation [9]. Sa posologie doit être augmentée en raison de l’augmentation des besoins en production de globules rouges [10].

L’aggravation de l’anémie est quasiment constante chez les femmes dialysées au cours de la grossesse. Elle est associée à une augmentation de l’incidence de la prématurité et à une mortalité infantile plus élevée [11]. L’urée en pré dialyse était supérieure à 14mmol/L chez toutes les patientes avec un taux moyen égal à 22,22mmol/L ± 4,29 mmol/L [14,66 - 28 mmol/L]. On n’a pas noté une corrélation ni entre le taux d’urée et la survenue d’hydramnios ni entre le taux d’urée et la MFIU. Ceci est probablement dû à la faible taille de l’échantillon. L’intensité de la dialyse et l’élimination de l’urée et des autres toxines urémiques joue un rôle essentiel dans le succès d’une grossesse établie. Avant la mise en œuvre généralisée de la dialyse, une étude a rapporté une corrélation directe entre la mortalité fœtale et les taux élevés d’urée. Une fois que les concentrations d’urée ont atteint plus que 21,4mmol/L, aucune naissance vivante n’a été documentée [7,12]. Dans notre étude, le volume horaire hebdomadaire de dialyse était ≥16 heures par semaine dans 7 cas et ≥20 heures dans 4 cas dans le but de diminuer les taux d’urée incriminés dans l’hydramnios. Nous avons trouvé une corrélation significative entre l’augmentation des heures de dialyse par semaine et la réussite de la grossesse (nouveau-né survivant au moins 28 jours) (R=0,59; p=0,002).

Chez les patientes en hémodialyse chronique, la fréquence d’HD doit être augmentée dès que possible à quatre séances puis à six séances par semaine à partir du cinquième mois pour optimiser le contrôle métabolique et de la volémie avec un objectif de 16 à 24 heures d’HD hebdomadaires qui est retrouvé associé à un meilleur pronostic fœtal [13]. En 2004, Hou a été le premier à démontrer une amélioration significative des naissances vivantes entre femmes sous hémodialyse recevant plus de 20h de dialyse par semaine pendant la grossesse [14]. La revue systématique et la méta-analyse de Piccoli *et al*. ont fourni un soutien supplémentaire au rôle de l’augmentation de la dialyse pendant la grossesse pour améliorer les résultats néonataux et, pour la première fois, fournir des preuves de l’hémodialyse comme modalité de choix pendant la grossesse [6,15]. En 2015, Piccoli examinait un grand nombre de séries de cas et de rapports de cas et indiquait que le nombre d’heures hebdomadaires HD était inversement corrélé avec l’accouchement prématuré (<37 semaines de gestation) et la naissance de petits bébés (inférieur au 10^ème^ centile). A ce titre, il devient de plus en plus courant d’augmenter considérablement l’intensité de la dialyse pendant la grossesse [6,7].

Concernant le diagnostic de grossesse, il est souvent retardé chez les femmes en âge de procréer en dialyse en raison de l’irrégularité des menstruations. Dans notre étude, l’âge moyen du diagnostic de grossesse était de 14 SA [3-30SA] alors qu’il est égal à 16 SA dans une série de cas marocaine publiée en 2015 [16]. Dans notre étude, le diagnostic de la grossesse a été suspecté devant un retard des menstruations dans 7 cas (28%), des signes sympathiques de la grossesse dans 6 cas (24%) et une augmentation du volume abdominal dans 2 cas (8%). Le diagnostic a été fait dans 9 cas (36%) par dosage des β-hCG, dans 9 cas par échographie seule (36%), par les deux dans 1 cas (4%) et non précisé dans 6 cas (24%). En effet, l’augmentation de la β-gonadotrophine chorionique humaine (β-hCG) est permanente en cas d’insuffisance rénale chronique, ce qui rend difficile le diagnostic de grossesse. Le test urinaire n’est pas valable, même chez les patientes non anuriques. C’est l’échographie pelvienne qui fera le diagnostic en cas de doute clinique, et précisera l’âge conceptionnel [17]. Les tests de β-hCG de sang en série et l’échographie pelvienne sont utiles pour confirmer la grossesse et déterminer l’âge gestationnel précis, plutôt que la date du dernier cycle menstruel, car les cycles irréguliers et l’anovulation sont fréquents dans l’IRC avancée. Dans notre série, l’âge gestationnel médian était de 34 semaines d’aménorrhée (28-38SA). Deux grossesses sont arrivées à un terme supérieur ou égal à 37SA. Les données rapportées dans le nouveau millénaire, décrivent un âge gestationnel médian de 33,8 semaines [18].

L’accouchement a eu lieu par césarienne dans 4 cas, et par voie basse dans les autres cas de notre série. L’induction planifiée du travail est recommandée à 37 semaines de gestation ou juste au-delà pour les patientes sans complications maternelles ou fœtales. Cette induction planifiée permet aux cliniciens de s’assurer que la patiente est bien dialysée. L’accouchement par voie basse est préférable et l’accouchement par césarienne est recommandé uniquement lorsqu’il existe une indication clinique claire. La planification et l’évaluation avancées par les équipes d’anesthésie et néonatales sont recommandées, compte tenu des besoins complexes de cette population de patientes. Dans notre expérience, le poids néonatal moyen est égal à 1970g ± 314g [1500g - 2300g] avec un âge gestationnel médian de 34 SA, qui est légèrement supérieur à celui de la littérature. Les données rapportées dans le nouveau millénaire, décrivent un âge gestationnel médian de 33,8 semaines avec un poids médian à la naissance de 1750g [19]. Parmi les complications maternelles, l’hypertension artérielle était la complication la plus fréquemment retrouvée dans notre étude (7 cas sur 20 si on élimine les 4 avortements précoces et l’IVG, soit 35% des cas).

Par ailleurs, aucun cas de pré-éclampsie, de diabète gestationnel ni de HELLP syndrome n’a été noté. Parmi les complications obstétricales, on a recensé 2 cas d’hémorragie de la délivrance. On n’a pas noté de complications de l’abord vasculaire à type de sténose, d’anévrysme ou d’infection au niveau de la fistule artério-veineuse, bien que dans la littérature une augmentation du risque d’anévrysmes au niveau de la fistule a été rapportée [20]. La pré-éclampsie et l’hypertension sévère sont les principaux facteurs de risque de prématurité et d’autres issues indésirables. Quatre-vingt pour cent des grossesses survenues chez les femmes en dialyse sont compliquées par l’hypertension qui est responsable de 1% de la mortalité des mères dans le passé. L’hypertension incontrôlée doit être traitée de manière adéquate, en maintenant la pression artérielle diastolique <80-90mmHg [21]. La pression artérielle systolique doit être maintenue entre 120 et 135 mmHg afin d’éviter l’ischémie placentaire secondaire à un défaut de la seconde invasion trophoblastique des artères spiralées du myomètre.

Deux complications obstétricales peuvent être rencontrées: l’hydramnios et la menace d'accouchement prématuré (MAP). Dans notre étude, l’hydramnios a été retrouvé dans 3 cas (16%). L’hydramnios est une complication obstétricale fréquente, peut être contrôlé par une augmentation de la fréquence et du temps d’HD [22]. La MAP est la survenue prématurée du travail responsable de la majorité des fausses couches tardives et des accouchements très précoces. Ces deux complications sont d'ailleurs étroitement liées, le polyhydramnios entrainant presque constamment l'apparition de contractions utérines. En fin, la grossesse a été considérée comme un succès si elle aboutissait à la naissance d’un nouveau-né survivant au moins 28 jours. Dans notre série, le succès de la grossesse était estimé à 56%. On note 2 décès dans les 28 jours après la naissance et 4 MFIU. Le taux de réussite des grossesses chez l’hémodialysée chronique varie selon les auteurs mais aussi selon les pays mais il semble aussi s’améliorer à travers les années avec des taux de succès arrivant jusqu’à 100% profitant ainsi des progrès des techniques d’hémodialyse [15].

## Conclusion

La grossesse au cours de l’insuffisance rénale chronique terminale est rare mais possible. La chance de donner naissance à un enfant vivant a augmenté de façon parallèle avec les progrès des techniques d’hémodialyse et la prise en charge néonatale précoce. Elles sont néanmoins considérées comme des gestations à très haut risque, et nécessitent à ce titre une surveillance accrue. L'équipe multi-disciplinaire qui prend en charge ces femmes est composée de néphrologues, d'obstétriciens, de néonatologistes et parfois de nutritionnistes qui travaillent en étroite collaboration. Quoi qu'il en soit, la préoccupation concernant la possibilité de devenir mère chez les jeunes femmes porteuses d'une maladie rénale chronique est presque constante. La patiente qui prend la décision de débuter une telle grossesse doit être armée d'une motivation personnelle très vive, en particulier en ce qui concerne la grossesse en dialyse, dont la prise en charge repose sur un protocole assez contraignant à suivre (un volume horaire hebdomadaire d’HD supérieur à 20h). De cette collaboration positive entre la femme et son équipe médicale référente, dépend la bonne qualité du suivi, et donc en partie l'augmentation des chances de voir naître un enfant en bonne santé. Assurer une hémodialyse intensive est une approche de traitement commune lors de la dialyse d'une femme enceinte. Les néphrologues préconisent maintenant 4 à 4,5 h par jour d’HD, 6 jours par semaine dès la confirmation de grossesse afin d’améliorer les résultats et diminuer le risque de survenue de complications maternelles et fœtales.

### Etat des connaissances sur le sujet

- La grossesse en dialyse est rare et à haut risque materno-fœtal;
- Une amélioration de la fertilité est possible avec les avancées technologiques en hémodialyse;
- Une intensification de la dialyse est nécessaire pour la réussite de la grossesse.

### Contribution de notre étude à la connaissance

- Une corrélation positive a été retrouvée entre l’augmentation du nombre de séances de dialyse et la réussite de la grossesse;
- Les complications materno-fœtales peuvent être minimisées grâce à une prise en charge étroite multidisciplinaire;
- Une attention particulière doit être donnée aux patientes dialysées en âge de procréation.

## Remerciements

Nous remercions tous les médecins des centres de dialyse du Sud Tunisien qui ont contribué à ce travail en envoyant leurs données et en répondant à toutes nos interrogations tout au long de ce travail.
